# “They treat us like rabid dogs”: Stigma and discrimination as experienced by people living with psychosis and their caregivers in Malawi—A photovoice study

**DOI:** 10.1371/journal.pmen.0000435

**Published:** 2025-09-15

**Authors:** Wakumanya Sibande, Dennis Chasweka, Kate Chidzalo, Demoubly Kokota, Eric Umar, Action Amos, Judith Allardyce, Angus Macbeth, Kazione Kulisewa, Annette Bauer, Michael Udedi, Charlotte Hanlon, Sumeet Jain, Stephen M. Lawrie, Lucinda Manda-Taylor

**Affiliations:** 1 Department of Health Systems & Policy, Kamuzu University of Health Sciences, Blantyre, Malawi; 2 Division of Psychiatry, Centre for Clinical Brain Sciences, University of Edinburgh, Edinburgh, Scotland, United Kingdom; 3 School of Health in Social Science, University of Edinburgh, Edinburgh, Scotland, United Kingdom; 4 Department of Psychiatry & Mental Health, Kamuzu University of Health Sciences, Blantyre, Malawi; 5 Department of Health Policy, London School of Economics and Political Science, London, United Kingdom; 6 NCDS & Mental Health Unit, Ministry of Health, Lilongwe, Malawi; 7 Department of Psychiatry, School of Medicine, College of Health Sciences, Addis Ababa University, Addis Ababa, Ethiopia; 8 Social Work Subject Area, School of Social and Political Science, University of Edinburgh, Edinburgh, Scotland, United Kingdom; PLOS: Public Library of Science, UNITED KINGDOM OF GREAT BRITAIN AND NORTHERN IRELAND

## Abstract

Psychosis in Malawi presents significant challenges for individuals living with psychosis and their caregivers, compounded by stigma, discrimination, and systemic barriers to healthcare access. This study employed a participatory photography method (photovoice) to explore the lived experiences of individuals with psychosis and their caregivers in the Chiradzulu and Salima districts. Sixteen participants, comprising eight People with Lived Experience (PWLE) of psychosis and eight caregivers, documented their experiences and perceptions through photographs. Photovoice participants reported stigma and discrimination in the form of social and economic exclusion and dehumanisation. Love, care, and self-reliance were identified and described as essential to recovery and combating exclusion. In contrast, healthcare access was described as both a facilitator and a challenge to recovery and caregiving. This study underscores the need for targeted community-based and policy-driven interventions to combat stigma and discrimination through intersectoral and multisectoral approaches. It also calls for integrated healthcare services such as task-sharing to address systemic barriers experienced by PWLE of psychosis and improved involvement of individuals with lived experience to improve recovery.

## Introduction

Mental health disorders significantly contribute to the global disease burden and greatly impact the quality of life for those affected. In 2022, mental health issues, including psychosis, were among the leading causes of Years Lived with Disability (YLDs), accounting for 125.3 million Disability-Adjusted Life Years (DALYs) worldwide [1]. In Malawi, over 70% of in-patients at the country’s tertiary mental hospitals are diagnosed with psychotic disorders [2]. Despite this high burden, its prevalence and lived experience of psychosis remain poorly understood at the community level, with no nationally representative prevalence studies to date.

Malawi’s mental health system is significantly under-resourced. Less than 1% of the national healthcare budget is allocated to mental health – far below the 15% recommended by the World Health Organization and the Abuja Declaration [1]. This underinvestment has translated into severely limited service provision, with only three consulting psychiatrists nationwide—all based in large urban centers, and poor engagement of people with lived experience of psychosis [3]. Moreover, People with Lived Experience (PWLE) of psychosis frequently experience exclusion from education, employment, and social welfare systems, and have little access to rehabilitation or livelihood programmes. Even basic needs such as food, clothing and shelter often go unmet, reflecting broader gaps in social protection and support [1].

Social and structural exclusion are further compounded by stigma and discrimination. Psychosis in Malawi is often misunderstood, with affected individuals facing social withdraw, family rejection, and community isolation. These experiences are not unique to Malawi but echo broader patterns seen in low- and middle-income countries (LMICs) [1].

To address these gaps, the Psychosis Recovery Orientation in Malawi by Improving Services and Engagement (PROMISE) project was designed to test and establish a community system for detecting and managing psychosis. This manualised intervention trains Health Surveillance Assistants (HSAs) in Malawi to screen for psychosis and refer individuals to appropriate care pathways [2].

The development of a culturally acceptable and contextually appropriate psychosis detection and management system requires close engagement with persons with lived experience (PWLE) of psychosis and their caregivers [1]. Using a participatory photography approach, known as photovoice [3], we aimed to understand better how psychosis is experienced by PWLE and caregivers in their communities. This collaborative process allowed us to explore personal narratives and diverse recovery pathways, ensuring the manualised intervention reflects the lived realities and cultural contexts of those affected by psychosis. Increasingly, there is a movement in the Global South to include and collaborate with individuals who have lived experiences of psychosis to address these challenges and improve care [4]. This involves taking those with lived psychosis experiences on board as co-creators and participants to avoid tokenism and ensure empowerment [5].

## Photovoice research

Initially employed in rural China to explore women’s reproductive needs, photovoice has since been adopted to address various public health issues [3]. As a method that recognises community members as participants, allowing them to capture experiences and perceptions that traditional research methods might overlook, it promotes a deeper understanding of participants’ lived realities, actions, and social change, enabling researchers to identify what participants appreciate about their lives and what needs to change. Photovoice empowers community participants to reflect on their challenges and successes through photos [3]. Participants take pictures that reflect their daily lives and provide narratives that can be used to effect social change in their communities. A defining characteristic of photovoice is its ability to prioritise the knowledge and experiences of those affected, facilitating their advocacy for addressing their social and health problems [4].

Photovoice is also considered user-friendly in low-income and literacy settings, as participants do not need expertise in photography or research experience. The method is also adaptable to diverse demographic groups. It can be applied to various social [3,6] and health issues, including palliative care for cancer patients [7], HIV in youth [8], and mental health [9,10]. For example, Cabassa et al. reported how photovoice was used to develop healthcare interventions among individuals with serious mental illnesses to prevent and treat cardiovascular diseases. PWLE and their caregivers have also used it to create mental health education and advocacy interventions [11].

Studies in high-income settings have also demonstrated that involving individuals with lived experience enhances understanding of nuanced issues and promotes further strengthening mental health systems to improve outcomes [12]. However, such involvement has been limited in low-income settings, sparking interest in using more participatory research methods to ensure meaningful inclusion [13].

In Malawi, photovoice has been employed across diverse contexts, including studies to explore treatment seeking among patients [14], and to address stigma and discrimination [15]. However, its application among individuals living with psychosis remains significantly underexplored, reflecting a gap in the literature on mental health research within this setting.

In this paper, we report how photovoice was used to explore and document the experiences and perceptions of PWLE and their caregivers and how these experiences influence the recovery and caregiving process. In the context of this research, the metaphor “rabid dogs” emerged from participant’s poignant descriptions of how individuals with mental illness are perceived and treated in their communities. In Malawi, rabid dogs are feared, avoided, and frequently stoned or killed on sight – a vicious reaction based on danger, fear, and stigma. The metaphor in this context was used to illustrate the intensity of dehumanising aspects of stigma and the pressing need to overcome such deeply ingrained societal attitudes by evoking this image. The photovoice study also formed part of the formative research for the PROMISE project.

## Materials and methods

### Ethics statement

This study was approved by the College of Medicine Research and Ethics Committee (P.03/23/4034) and the University of Edinburgh Medical School Research Ethics Committee (22-EMREC-044). The participants were provided with participant information sheets and verbal explanations, and they gave written consent through either a thumbprint or signature. They also provided informed consent to have their pictures used in this publication and were also given private property and photography release forms to administer to their research subjects. These forms ensured that individuals appearing in the photographs used in this study gave their explicit consent to be photographed and published.

### Study design

This study employed a qualitative participatory action design, using a phenomenological approach to collect and analyse data [16]. Data was collected through participant-taken photographs, individual interviews, and group discussions. This approach facilitated participants’ active involvement as co-producers of research evidence, allowing them to capture and share their experiences and perceptions of psychosis. Phenomenology provided a framework to explore and interpret participants’ lived experiences and perspectives, offering rich insights into the personal and social dimensions of living with psychosis.

### Study setting

Malawi, a landlocked country between Central and Southern Africa, has an estimated population of 21.3 million at last estimate. [17]. The country’s economy is predominantly agricultural, with approximately 80% of the rural population engaged in subsistence farming. The study was carried out in two districts in Malawi: Chiradzulu, a rural district situated in the southern region, approximately 26.7 kilometres northeast of Blantyre City, and Salima, a largely rural district with a semi-urban town located in the central region, about 267.5 kilometres from Blantyre.

### Description of participants

The study involved PWLE of psychosis and their caregivers. All PWLE participants had previously received a clinical diagnosis of a psychotic disorder from mental health professionals at a tertiary healthcare facility (psychiatrist) or at a secondary healthcare facility (psychiatric clinical officer). While the study did not aim to clinically assess symptom severity at the time of participation, all the selected participants had a history of psychotic symptoms, including visual and auditory hallucinations, delusions, disorganised thinking and social withdrawal. At the time of the photovoice research period, all the participants included in the study were in recovery phases, with different degrees of functionality and support needs. Individuals presenting with moderate-severe to severe psychotic symptoms at the time of recruitment were withdrawn from the study to protect their well-being. Alongside PWLE were their caregivers. These were either parents, siblings, spouses, or friends of the PWLE.

### Participant recruitment, sample size, and data collection methods

The recruitment, enrolment, and consent procedures for the photovoice study, which involved identifying PWLE of psychosis and their caregivers, were conducted purposively in consultation with the mental health clinicians at Chiradzulu and Salima District Hospitals and focused on those from villages surrounding Chiradzulu and Salima District Hospitals. The inclusion criteria required participants to be between 18 and 65 years old, with a lived experience of psychosis not attributable to an organic cause such as epilepsy or HIV. Participants were excluded if they were not residing in the two districts or were acutely psychotic and unable to decide on participation. Voluntary written informed consent was obtained from all participants before participating in the study. There were no established relations between the researchers and the study participants in either district, and all participants met the researchers for the first time through this study.

At the start of the study, 12 participants were sampled in Chiradzulu, consisting of six pairs, each comprising an individual with lived experience of psychosis and their caregiver. One pair was excluded for arriving late to the initial photovoice orientation workshop, and they were compensated for their time and their transport reimbursed. In Salima, 12 participants were initially sampled, but three pairs were excluded due to being under 18, not having psychosis, and lacking spousal consent. The final sample comprised 10 participants in Chiradzulu (five pairs) and six in Salima (three pairs). The total sample size was 16 participants (eight pairs).

During the first sessions (on 29^th^ May 2023 in Chiradzulu and 14^th^ August 2023 in Salima), potential participants were invited to a day-long workshop where they were informed about the PROMISE project, their permission to join in the photovoice research was requested and informed consent was obtained. Additionally, participants were trained on using Photovoice as a participatory photography method, specifically using a smartphone to take and store photographs. The training also covered photography ethics, including obtaining written informed consent from individuals or capturing identifiable features (such as buildings or property) that may appear in the photographs that belong to individuals.

The second session occurred a week later (7^th^ June 2023) in Chiradzulu and two weeks later (16^th^ August 2023) in Salima. Participants were invited to share their successes and challenges with the participatory photography method. Additionally, the sessions involved checking how they documented informed consent forms to ensure compliance with ethical standards.

After twenty-one days, the third session (19^th^ June 2023 in Chiradzulu and 18^th^ September 2023 in Salima) involved inviting the participants to a workshop, where we discussed the photographs using adaptations to the SHOWeD technique [18], which modifies the original prompts with the PHOTO technique asking a series of reflective questions designed to deepen participants’ engagement with the images and their experiences. The questions posed during discussions included: 1) Describe your **P**icture. 2) What is **H**appening here? 3) Why did you take a picture **O**f this? 4) What does this picture **T**ell us about your life? 5) How can this picture provide **O**pportunities to improve life for the population of interest? [19]. Through this process, participants could describe their experiences and highlight what actions stakeholders must take to support the recovery of PWLEs. At the end of the discussion, each participant chose five photos of the most significant for discussion with the other participants during the fourth session.

**Fig 1:**
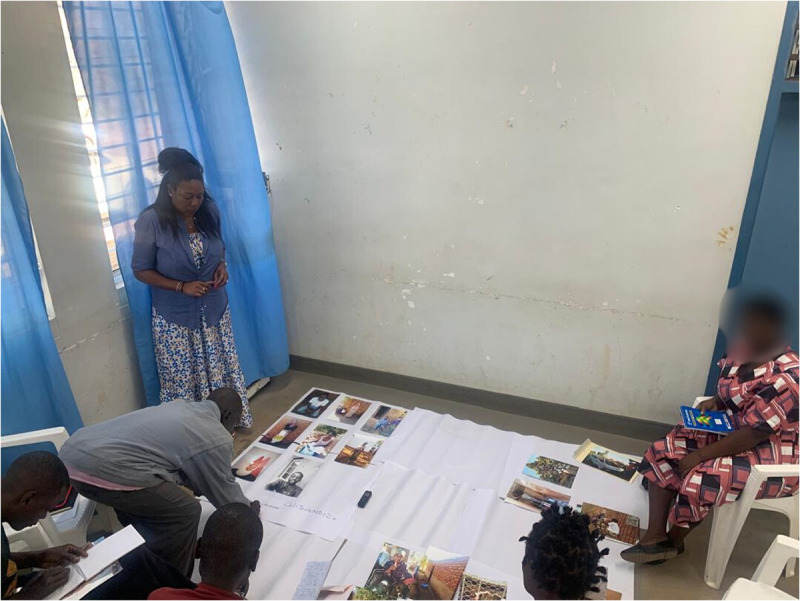
Photovoice participants (PWLE) sorting and categorising photographs into initial themes facilitated by researchers.

**Fig 2:**
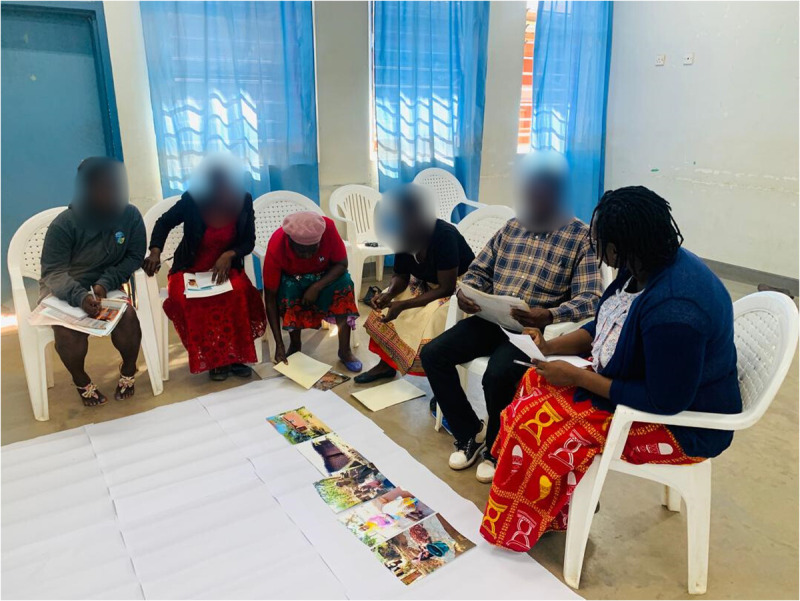
Photovoice participants (Caregivers) sorting and categorising photographs into initial themes facilitated by researchers.

The fourth session (20^th^ June 2023 in Chiradzulu and 19^th^ September 2023 in Salima) was a focus group discussion to which all study participants in each district were invited. For the group discussions, PWLE were in one group, and the caregivers were in a separate group. Each participant’s photos were laid on the floor for the group to see. The participants were instructed to explain their pictures to the rest of the group – why they took them and how it relates to their everyday life. After introducing each photograph, other group members were encouraged to discuss the picture. Participants grouped photos with similar meanings into themes, and the participants were asked to provide a caption describing the grouped photos. Then, each group ranked the top three themes representing priority issues they wanted to communicate to community and district stakeholders. The fifth and final sessions (7^th^ July 2023 in Chiradzulu and 25^th^ September 2023 in Salima) were workshops that brought together community and district stakeholders for participants to share the findings of the study, express their desires and needs, and invited stakeholders to respond, provide feedback, and comment on the findings Figs 1 and 2.

### Data analysis procedures

#### Participant involvement.

As described above, the initial discussions involved reviewing the photographs using the PHOTO technique and asking PWLE to choose the top five photographs closely related to their daily lives. Once individuals selected their top five photographs, the PHOTO technique was used again to revisit and explore them. These questions enabled participants to identify and prioritise the pictures that best captured their lived experiences, allowing for a deeper exploration of the broader social, cultural, and personal contexts of the issues presented. The selected five photographs were further discussed using the PHOTO technique during the audio-recorded interviews.

#### Theme development.

In the group discussions, the study team facilitated grouping photographs based on commonly agreed meanings, generating captions that informed the initial themes. Through this process, participants were also able to articulate their experiences and highlight the actions stakeholders must take to support the recovery of PWLE. All individual and group discussions were facilitated by five (two females and three males) researchers, LMT, KC, WS, DK, and DC, who are all experienced in conducting qualitative interviews.

All interviews and group discussions were conducted face-to-face and in the local language of Chichewa, were audio-recorded, transcribed verbatim, and subsequently translated into English. Completing both the individual and group discussions took the entirety of one morning. The analysis of the photographs took place during the group and individual discussions. Thematic analysis techniques were used to analyse pictures and transcripts.

#### Coding.

Transcripts were then coded by two experienced social scientists, LMT and WS. Divergences between coders were resolved through discussion, leading to developing two codebooks—one for PWLE and one for caregivers. Data was subsequently coded in NVivo14 software by WS and organised into overarching themes and sub-themes. Initially, separate themes were developed for PWLE and separate themes for caregivers, but after thorough reorganisation, comparison, and categorising, overlapping themes were merged and reported in our findings.

#### Analysis.

Data were analysed using a modification of the three-stage participatory group analysis developed by Wang and colleagues [3,20]. The three phases are selecting photographs that most accurately reflect the participants’ views, contextualising the pictures, and codifying emerging issues, themes, or theories. As described above, the participants completed phases one and two in sessions three and four. WS & LMT collaborated to complete a qualitative analysis for phase three. A theme, identified as a concept that emerged from a participant’s written or spoken expression, included secondary themes after reorganising, comparing, categorising, and grouping the common themes from the participant’s selected photos, their descriptions, captions, and transcripts. These data sources were grouped to structure the analysis and presentation of the findings [21]. This process allowed us to ensure that our analysis empowers our participants and is critically reflective by enabling participants to identify their concerns and articulate their needs.

### Researchers’ positionality

The researchers in this photovoice study were mindful of the potential influence of their biases, assumptions, and beliefs on the research approach and the interpretation of participants’ experiences. Three of the researchers (DK, DC, & KC) had prior experience working with PWLE and caregivers, granting them a nuanced understanding of the challenges faced by participants. Even though this was beneficial, they consciously tried to remain receptive to participants’ unique perspectives and avoid over-reliance on prior knowledge. The other two researchers (LMT & WS) have experience undertaking health systems research in Malawi, including work within the Salima district, one of the study locations. In mitigation, researchers actively sought new insights, knowledge, and evidence, remaining open to participants’ voices. This reflexive approach allowed the researchers to make interpretations that reflected the context of the participants in the photovoice study.

### Credibility and trustworthiness

To ensure rigour in our analysis, we employed researcher triangulation by involving multiple researchers in the coding and interpretation of data, each bringing different disciplinary perspectives. Second, to improve credibility, a subset of coded transcripts were reviewed collaboratively by WS, LMT, and DC, and discrepancies were resolved through discussion. Researchers in this photovoice study were not blinded to participants’ roles due to the nature of the interviews and the need to retain contextual understanding during interpretation.

## Results

### Participants demographic information

Table 1 illustrates an equal distribution of male and female participants among persons with lived experience (PWLE) and caregivers. Most participants identified as Christian (n = 14), as the study districts are predominantly Christian. Regarding educational background, most had completed primary education (n = 8) and obtained a Primary School Leaving Certificate (PSLCE), with a quarter having attained secondary education (n = 6) with either a Junior Certificate of Education (JCE) or a Malawi School Certificate of Education (MSCE). Only one participant completed their tertiary education.

**Table 1:** Participants Demographic Information.

Variable	Values	Frequency
Age	< 40	7
	> 40	9
	Median	44.3
	Range	18-65
Sex	Male	8
	Female	8
Role	Person with lived experience	8
	Carer	8
Religious affiliation	Islam	2
	Christian	14
Education	Tertiary	1
	MSCE	4
	JCE	1
	PSLCE	8
	Primary	2

Our findings, as summarised in Table 2, are organised into three major themes that describe participants’ experiences of psychosis: first, the stigma and discrimination faced by PWLE of psychosis and their caregivers; second, the lived experiences and needs; and third, a description of their encounters with accessing healthcare services. We followed the Consolidated Criteria for Reporting Qualitative Research checklist (COREQ) [22].

**Table 2:** Summarised key themes and representative quotations.

Theme	Description	Representative Quotes
Stigma and discrimination among PWLE and caregivers: exclusion, abandonment, and prejudice	Participants described how their mental health status leads to social and economic exclusion, abandonment, and dehumanisation	*“Because we are mentally ill, they (Traditional leaders) don’t include us when registering for fertiliser coupons. We should tell the chiefs that they should include us when they register for fertiliser coupons.” (Male PWLE, FGD Participant 02. Chiradzulu)* *“There is also another form of discrimination where a person is abandoned by their parents; they just say, leave this one alone... Sometimes, there is abuse that parents inflict on their children, like when the child wants to go to school, they say no, you must not go to school.” (Male PWLE, FGD Participant 07. Salima)* *“They treat us like dogs with rabies. Dogs are discriminated against; they eat leftovers, so we wonder why we are discriminated against as people suffering from psychosis, which is wrong.” (Male PWLE, FGD Participant 05. Chiradzulu)* *“The ones that suffer from HIV/AIDS, diabetes, and TB. If they go today, they will find their medication. But if we go, they will tell us there is no medication for the district hospital. Going to the district hospital, they also tell us that there is no medication, and they say if you have money, go and buy it at a pharmacy.” (Male PWLE, FGD Participant 08. Salima)*
Love, care, and autonomy: key dimensions of support for individuals with psychosis	PWLE and caregivers provided insights into the relevance of love, care, and autonomy in the recovery process for PWLE and caregiving for caregivers. They described practical support from family, friends and caregivers to constitute love and care. Self-reliance encompassed autonomy and agency.	*“The first thing we can do is to monitor our patients so that when the illness starts, we should rush them to the hospital. But even before the illness starts, we should encourage the patient to take their medication every day and accordingly. This way, we will not reach a point where the patient falls seriously ill.” (Male Caregiver FGD Participant 01. Chiradzulu).* *“Okay. I always admire that if I can improve with my boss right…because I can do things like measuring maize. So, I think to myself that if I can find my capital and open my shop and assist people with psychosis, it is going to benefit those with psychosis” (Male PWLE. IDI Participant 05. Chiradzulu)* *“As you have said, this research is about mental illness, so the relationship is this: we are patients of psychosis, but we can think as such, we need to take care of ourselves; for example, if you lack clothes and you get some money, you should be able to buy clothes. If you don’t have money, ask for it from someone who can help you. On top of that, if we get money, we should be able to buy medicine for ourselves.” (Male PWLE. IDI Participant 07. Salima)* *“As caregivers, we should work hard by having at least something to generate income, like small businesses. Business doesn’t mean it has to be something big. Even small-scale businesses are important. But also, patients should not underrate themselves because others rely on being helped only. Patients should have a self-help mentality. If caregivers participate in an income-generating activity, the patients should assist them in improving their livelihood.” (Male Caregiver, FGD Participant 01. Salima)*
Access to Healthcare services: facilitators and barriers to recovery	Participants highlighted the importance of timely and convenient access to healthcare services as essential for managing psychosis and supporting recovery. Their failure to access healthcare services acts as a barrier to recovery and optimal caregiving.	*“From what I have seen, what is needed is the availability of clinics near our communities because our patients won’t face challenges in accessing care, and drugs should not be scarce. If drugs are available, even for us caregivers, our lives will improve just like the patients, we will all be happy.” (Female Caregiver. IDI Participant 02. Salima)* *“Another thing is that it happens that you travel that distance and get there, then they tell you that there is no medication, and for you to go again the following day, you are usually lazy because you know that you will not find medicine at the hospital, so you say I would rather be home when you know that you are sick.” (Male PWLE. FGD Participant 07. Salima)*

### Stigma and discrimination among PWLE and caregivers: Exclusion, abandonment, and prejudice

Under this theme, discrimination against PWLE was described as manifesting in multiple, interrelated ways, affecting their participation in social, economic, and educational activities. Participants in this study described how their mental health status leads to exclusion, abandonment, and prejudice, perpetuating cycles of poverty, isolation, and diminished self-worth. Exclusion was a recurring experience for PWLE, particularly in economic activities vital to rural livelihoods. Participants expressed frustration at being intentionally sidelined from community initiatives and excluded from the government of Malawi support programs, including those subsidising farmers with ‘fertiliser coupons’. This exclusion from critical agricultural programs not only perpetuates cycles of poverty and dependency for PWLE, but also undermines their ability to contribute to household productivity and improve their self-worth, further isolating them socially and economically. This further exacerbates food insecurity and financial instability, which are closely linked to poor mental health outcomes:

*“Because we are mentally ill, they (Traditional leaders) don’t include us when registering for fertiliser coupons. We should tell the chiefs that they should include us when they register for fertiliser coupons.” (Male PWLE, FGD Participant 02. Chiradzulu)*

Abandonment emerged as another distressing form of discrimination. PWLE shared experiences of being neglected by their families, with some parents actively denying them access to education. For caregivers and PWLE, the absence of family support deepens their isolation and vulnerability:

*“There is also another form of discrimination where a person is abandoned by their parents; they just say, leave this one alone... Sometimes, there is abuse that parents inflict on their children, like when the child wants to go to school, they say no, you must not go to school.” (Male PWLE, FGD Participant 07. Salima)*

**Fig 3:**
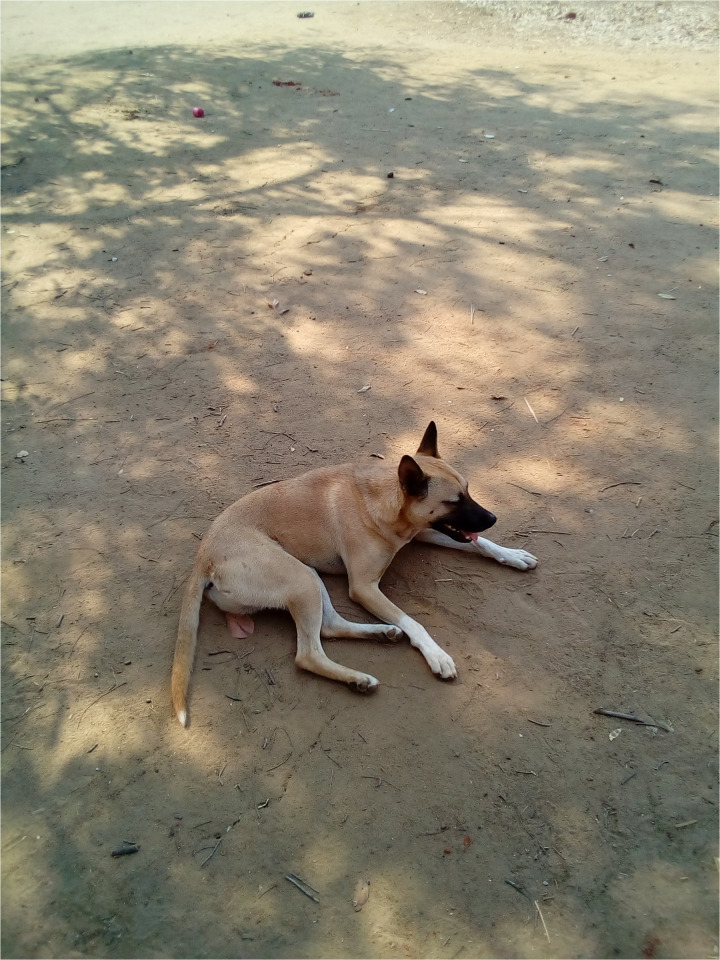
This photograph was taken by a PWLE who reflected on it as a reminder of how people in their community discriminate against them and dehumanise them through derogatory name-calling.

Being denied the opportunity to access education limits their participation in the formal and informal economy and deprives PWLE of social engagement, further undermining their self-esteem. The emotional harm caused by abandonment leaves PWLE feeling like a burden to both their families and society at large Fig 3.

Stigma, discrimination, and dehumanisation faced by PWLE are vividly captured in both personal experiences and symbolic imagery. One participant in this study used the metaphor of a dog to illustrate how society strips PWLE of their dignity and humanity through derogatory name-calling. In his account, being likened to a stray or rabid dog reflects how PWLE are treated as outcasts, reinforcing both social stigma and emotional distress:

*“They treat us like dogs with rabies. Dogs are discriminated against; they eat leftovers, so we wonder why we are discriminated against as people suffering from psychosis, which is wrong.” (Male PWLE, FGD Participant 05. Chiradzulu)*

The photograph of the dog, taken by the participant, visually represents how community members marginalise PWLE through exclusionary behaviour. Participants reported that such dehumanisation contributes to the erosion of their self-worth, further isolating them from social and economic life.

Participants also expressed frustration over how their needs are not prioritised within the healthcare system, particularly in comparison to individuals with other chronic conditions such as HIV/AIDS, diabetes, and tuberculosis (TB). PWLE recounted experiences of the unavailability of antipsychotic medication, underscoring their perceptions of healthcare discrimination and neglect within the health system.

*“The ones that suffer from HIV/AIDS, diabetes, and TB. If they go today, they will find their medication. But if we go, they will tell us there is no medication for the district hospital. Going to the district hospital, they also tell us that there is no medication, and they say if you have money, go and buy it at a pharmacy.” (Male PWLE, FGD Participant 08. Salima)*

This quote illustrates how psychosis is often seen as a lower priority in healthcare settings. The lack of urgency in treating these conditions leaves PWLE feeling abandoned and unworthy of the care and support extended to others with different health challenges. This systemic neglect further exacerbates their exclusion and alienation, reinforcing a cycle of marginalisation that impacts both their mental and physical well-being.

### Love, care, and autonomy: Key dimensions of support for individuals with psychosis

Participants in this study shared profound insights into the importance of love and care in the recovery process for PWLE. Caregivers and PWLE highlighted how emotional support, practical caregiving, and meaningful relationships contribute to well-being. Alongside love and care, the participants emphasised autonomy and agency as essential factors in empowering individuals with psychosis to regain control over their lives and manage their condition independently. These two sub-themes—Love and Care and Autonomy and Agency—capture the emotional and practical elements that shape the experiences of PWLE and their caregivers.

#### Love and care.

Both PWLE and caregivers identified love and care as essential for fostering recovery and improving well-being. PWLE emphasised that love is demonstrated through acts of care, such as ensuring access to medication and basic needs. Family members, friends, and community members were highlighted as key sources of support, providing practical help and emotional encouragement.

*“We find love through our parents. Some parents notice that their child’s medicine is about to be finished, so they should buy more. Even in churches, they say we have found this person. He is sick. Let us pray for him. That is love.” (Male PWLE. FGD Participant 07. Salima).*

Similarly, caregivers shared how caring for PWLE involves monitoring them, ensuring medication adherence, and promptly responding to signs of illness. They explained that caregiving requires attentiveness to their evolving needs and collaboration with healthcare services to avoid severe episodes.

*“The first thing we can do is to monitor our patients so that when the illness starts, we should rush them to the hospital. But even before the illness starts, we should encourage the patient to take their medication every day and accordingly. This way, we will not reach a point where the patient falls seriously ill.” (Male Caregiver FGD Participant 01. Chiradzulu).*

Participants also reflected on the importance of family, friendships, religious groups, and healthcare providers in promoting recovery and preventing stress. These supportive relationships provide practical help, emotional resilience, and social support for PWLE. Caregiving relationships described by participants also reflect principles of the stress-buffering hypothesis, where consistent support acts as a protective factor against the psychological impacts of exclusion and discrimination.

*“My mental illness started because of depression after my marriage ended with my former wife. So when I meet people like him, they help me not to be depressed.” (Male PWLE IDI Participant 08. Chiradzulu).*

**Fig 4:**
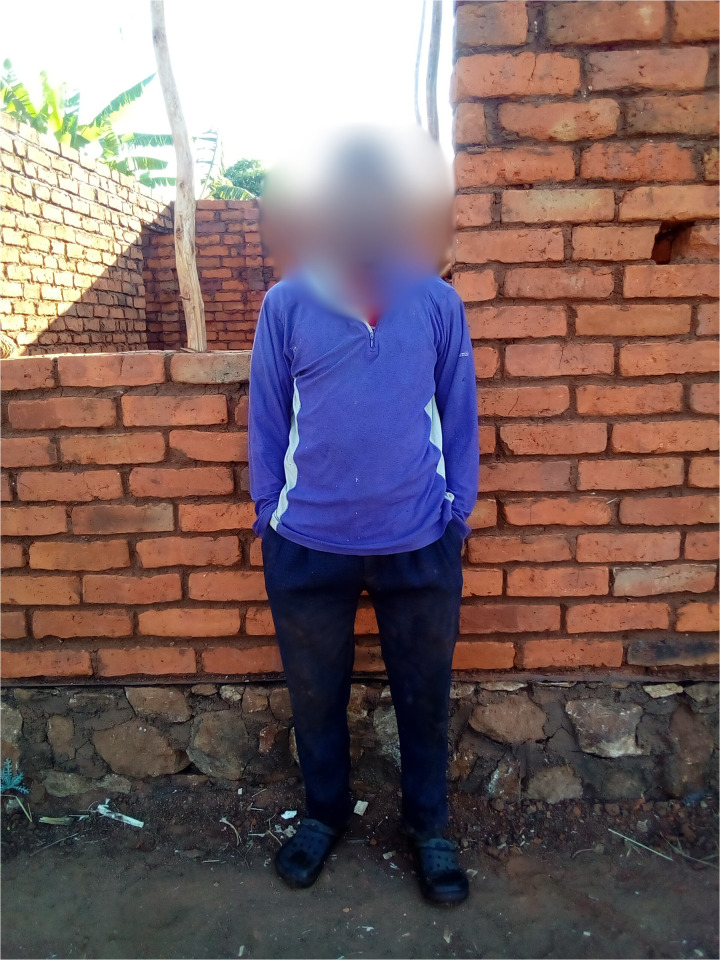
This photograph was taken by an individual living with psychosis. The person in the picture is a friend of the individual with psychosis and was taken to reflect on one of his sources of love and care as a patient, which helps him not to be stressed and worried.

**Fig 5:**
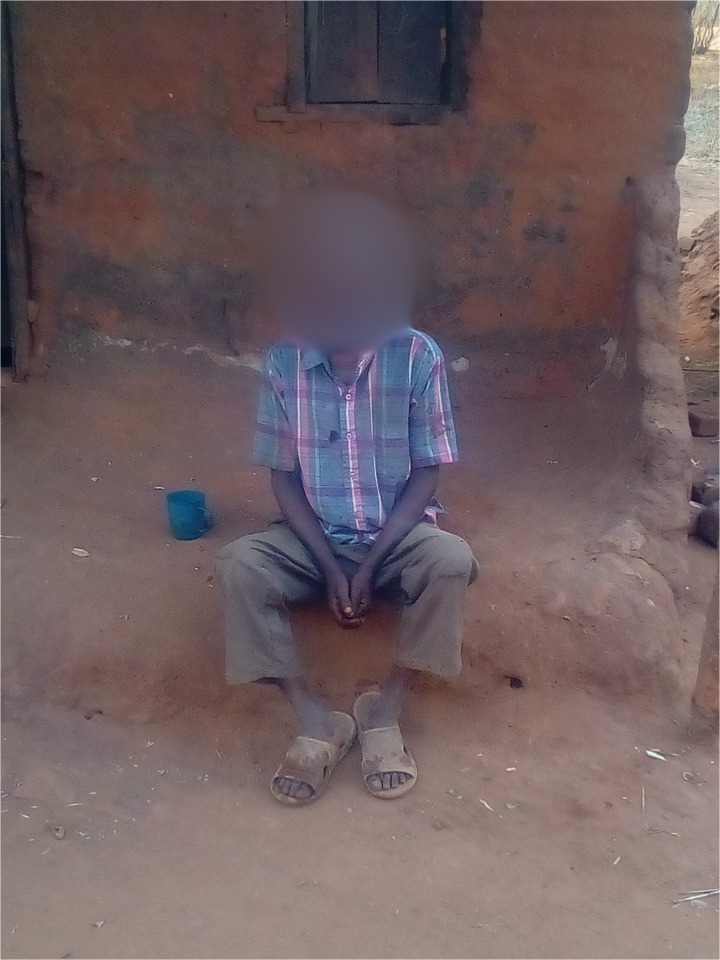
This photograph depicts an older person who is one of the caregivers. The photo was taken to reflect the idea that caregiving is supposed to be everyone’s role in the community.

Caregivers also reflected on the importance of collective responsibility in caregiving. They emphasised that caregiving should not be restricted to gender or age but should involve the entire community Figs 4 and 5:

*“I want all of us to have unity and affection towards PWLE to defeat this disease. This is by not sparing anyone, be it village chiefs or employed people; all of us should have affection towards PWLE; that is very important. Without love, there is no peace. If we work hand in hand, there won’t be discrimination.” (Female Caregiver. FGD Participant 01. Salima).*

#### Self-reliance and autonomy.

In addition to love and care, participants also highlighted the importance of self-reliance and autonomy in supporting individuals with psychosis. Self-reliance and autonomy emerged as vital elements in PWLE’s recovery journey. Participants emphasised the importance of managing their illness independently and meeting daily needs without excessive reliance on others. The ability to engage in income-generating activities—such as employment and small businesses—was identified as a key factor in fostering empowerment, contributing to both personal and economic well-being. Autonomy was crucial in restoring self-worth and controlling their lives, which participants believed was central to recovery.

*“Okay. I always admire that if I can improve with my boss right…because I can do things like measuring maize. So, I think to myself that if I can find my capital and open my shop and assist people with psychosis, it is going to benefit those with psychosis” (Male PWLE. IDI Participant 05. Chiradzulu)*

Participants described autonomy as making decisions, managing resources, and meeting their needs, which extended beyond economic independence to encompass taking control of their healthcare, including purchasing medication.

*“As you have said, this research is about mental illness, so the relationship is this: we are patients of psychosis, but we can think as such, we need to take care of ourselves; for example, if you lack clothes and you get some money, you should be able to buy clothes. If you don’t have money, ask for it from someone who can help you. On top of that, if we get money, we should be able to buy medicine for ourselves.” (Male PWLE. IDI Participant 07. Salima)*

**Fig 6:**
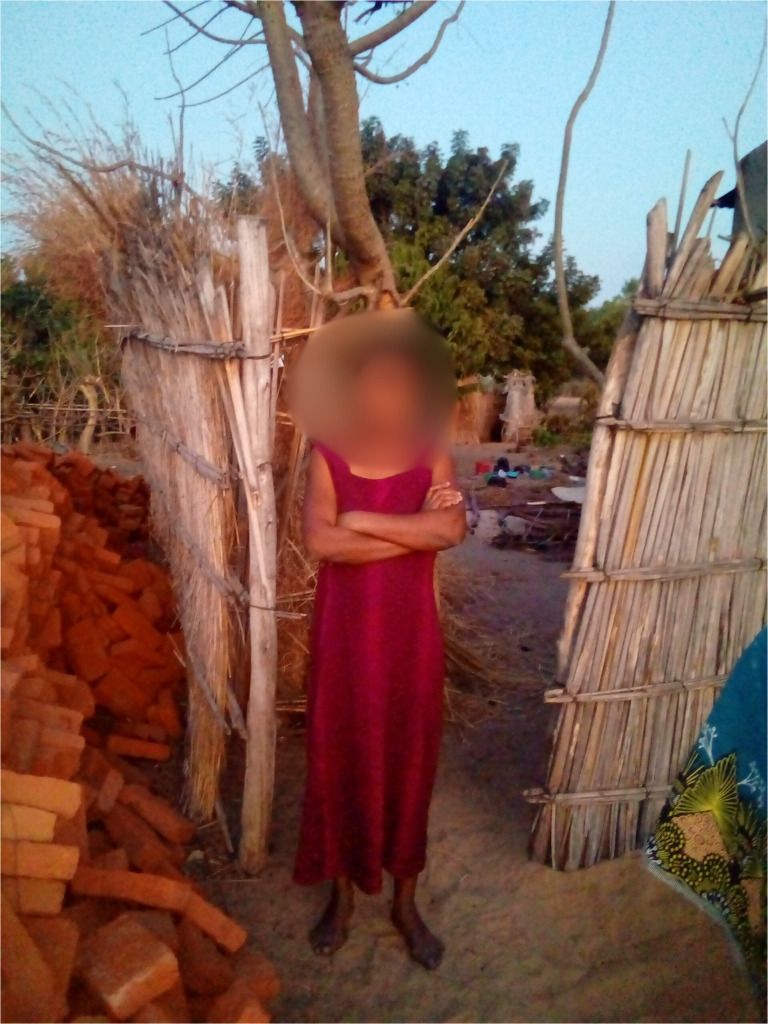
This photograph was taken by a PWLE. It was used to reflect self-efficacy and autonomy. The participant reflected that the girl in the picture has psychosis, yet she is self-reliant, which enables her to support herself throughout the illness, a thing PWLE desire.

**Fig 7:**
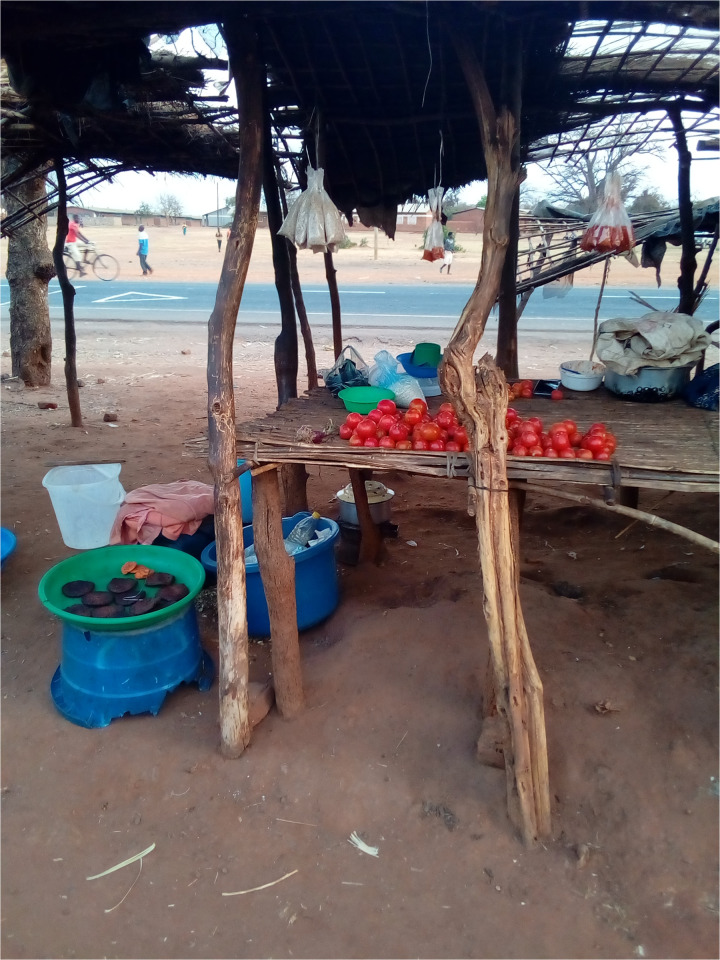
A caregiver reflected on this photograph of a business as a symbol of self-reliance. Caregivers desire to be self-reliant and have a stable household income to care for their patients.

Caregivers discussed how generating household income through small businesses is essential for maintaining optimal caregiving. They noted that shared participation in economic activities benefits caregivers and patients, fostering collaboration and promoting independence for both parties Figs 6 and 7.

*“As caregivers, we should work hard by having at least something to generate income, like small businesses. Business doesn’t mean it has to be something big. Even small-scale businesses are important. But also, patients should not underrate themselves because others rely on being helped only. Patients should have a self-help mentality. If caregivers participate in an income-generating activity, the patients should assist them in improving their livelihood.” (Male Caregiver, FGD Participant 01. Salima)*

This perspective emphasises that caregiving is most effective when caregivers and PWLE cultivate a mindset of self-reliance, actively contributing to their well-being and that of the household. However, self-reliance and autonomy among PWLE and their caregivers are constrained by some external factors such as exclusion and poverty.

### Access to healthcare services: Facilitators and barriers to recovery

Access to healthcare emerged as a critical theme in the experiences of PWLE and their caregivers. Participants highlighted the importance of timely and convenient access to healthcare services as essential for managing psychosis and supporting recovery. However, they also described multiple barriers—such as long distances to healthcare facilities and the unavailability of essential medications—that complicate caregiving and contribute to relapses. This theme underscores how access to healthcare affects patient outcomes and influences caregivers’ quality of life.

Many participants emphasised that long distances to health facilities and frequent stockouts of antipsychotic medication at district hospitals were among the most significant challenges they faced. These barriers discouraged regular visits to healthcare facilities and delayed treatment, increasing the risk of relapses and delaying recovery.

*“From what I have seen, what is needed is the availability of clinics near our communities because our patients won’t face challenges in accessing care, and drugs should not be scarce. If drugs are available, even for us caregivers, our lives will improve just like the patients, we will all be happy.” (Female Caregiver. IDI Participant 02. Salima)*

This quote reflects the frustrations of people caring for PWLE, who often find themselves discouraged by repeated failed attempts to obtain medication, resulting in feelings of helplessness.

Caregivers noted that proximity to healthcare services and reliable access to medicines were key facilitators of caregiving. They expressed that having health facilities or services closer to the community would ease the burden on people with long-term epilepsy (PWLE) and their caregivers, ensure continuous access to care, and improve overall well-being.

*“Another thing is that it happens that you travel that distance and get there, then they tell you that there is no medication, and for you to go again the following day, you are usually lazy because you know that you will not find medicine at the hospital, so you say I would rather be home when you know that you are sick.” (Male PWLE. FGD Participant 07. Salima)*

**Fig 8:**
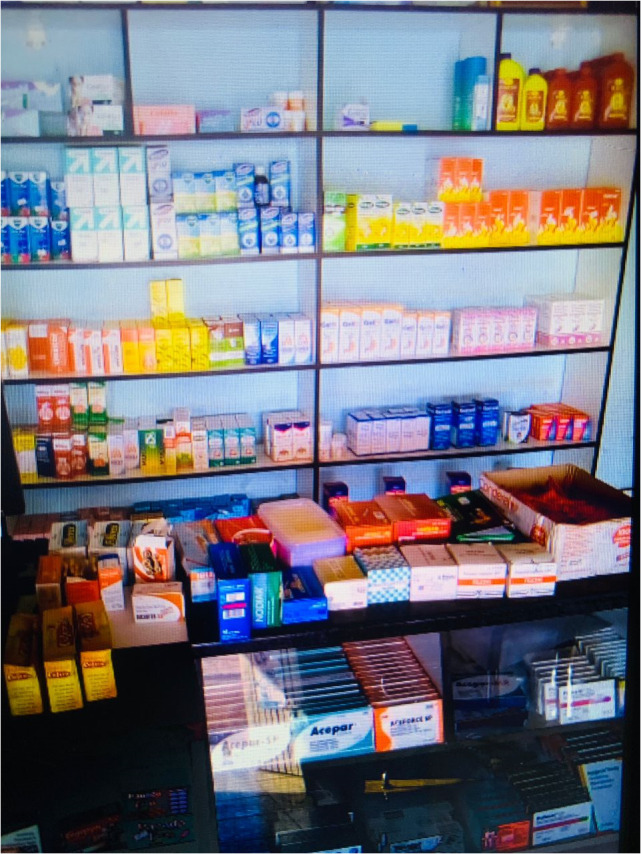
An individual with psychosis took this photograph. It is a photo of a local pharmacy where he buys his medicine when the district hospital is out of stock. It reminds him of the existing systemic barriers to psychosis care.

**Fig 9:**
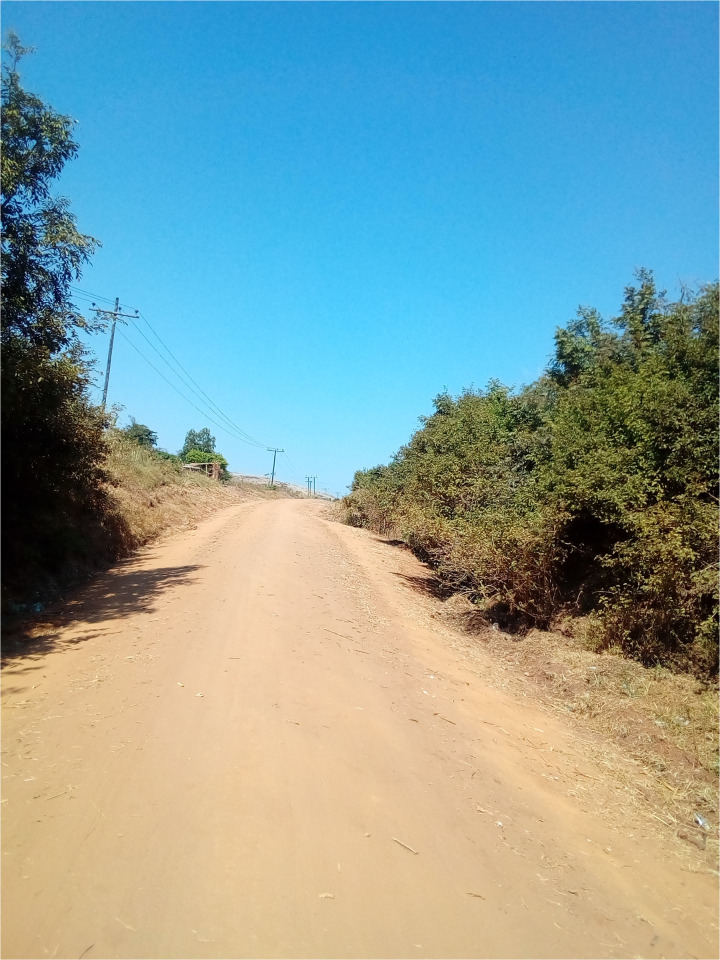
An individual living with psychosis reflected on how long it takes to get to the hospital using this road, and it reminds him of how he and other PWLEs struggle to access psychosis care.

This perspective highlights how healthcare access affects patients’ health outcomes and directly influences caregivers’ ability to provide adequate care and maintain their well-being Figs 8 and 9.

### Illustration 1: A conceptual illustration of the key themes and their interconnection


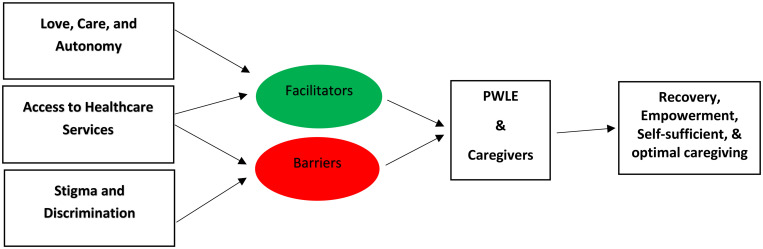


As illustrated above, the key themes identified in this study function as facilitators, barriers, or in some cases, both, in relation to the recovery of PWLE of psychosis and the capacity of caregivers to provide optimal support. Love, care, and autonomy promote both recovery and effective caregiving. Access to healthcare services emerged as a critical enabler; however, it’s absence or limited availability constituted a significant barrier to positive patient outcomes. Additionally, stigma and discrimination were consistently identified as impediments to both recovery and provision of optimal caregiving.

## Discussion

To our knowledge, this study is the first to use photovoice to explore and document the experiences and perceptions of PWLE of psychosis and caregivers in Malawi. This approach enabled participants to reflect on their personal experiences and identify what is important to them. The participatory nature of photovoice allowed participants (PWLE and caregivers) to express factors that positively or negatively affect recovery and the provision of care. Three main themes were identified from participants’ accounts of living with or caring for people with psychosis. The findings are interpreted and discussed through the lens of stigma theory, particularly Goffman’s conceptual model, which views stigma as the co-occurrence of labeling, stereotyping, separation, status loss, and discrimination [23,24].

The first theme, stigma and discrimination, highlights deeply rooted experiences of marginalisation within social and economic structures which drive PWLE and their caregivers into poverty and isolation. These findings align with other studies and theoretical frameworks that have shown how stigma and discrimination negatively impact medication adherence, quality of life, and social functioning among PWLE and caregivers [25,26]. Similarly, social and economic isolation, along with discrimination in healthcare settings, has been documented in previous research [27], including sectors such as education, social welfare, housing, and employment [1], which our photovoice study has also observed. According to Goffman, such stigma emerges through every day social interactions where individuals are marked as different and become subject to social distancing and avoidance. In our study, this was reflected in participants’ accounts of being excluded from community life and treated as less human.

While much of the existing global evidence emphasizes the psychological burden of social stigma, our findings suggest that stigma and discrimination also restrict access to economic opportunities, including exclusion from state social programs such as farm input subsidies and employment opportunities. This exclusion compounds economic inequalities, further hindering personal and social recovery. Similar findings have been reported in studies from Ethiopia, where structural discrimination creates barriers to both healthcare access and economic participation [28,29]. Recovery from severe mental illness requires intersectoral approaches, particularly through inclusion in development programs, as these initiatives are crucial for improving economic stability and supporting long-term recovery [30]. Without access to such programs, PWLE remain excluded from opportunities that are critical to achieving personal and social recovery, perpetuating cycles of poverty and marginalisation [31]. Such systemic exclusion is consistent with Goffman’s observation that stigma not only affects personal identity, but also limits access to valued social roles, thereby reinforcing disadvantage and dependency.

The themes describing participants’ need for love and care, self-reliance and autonomy reflect the dual importance of emotional and practical support, alongside personal empowerment in the lives of individuals living with psychosis. Participants emphasised that love and care, expressed through supportive relationships and caregiving, lay the foundation for recovery. At the same time, promoting self-reliance and autonomy enables PWLE to regain control over their lives and manage their condition effectively. The community’s role in fostering care and independence is essential in breaking cycles of discrimination and facilitating meaningful recovery. This reinforces the value of empowering PWLE and caregivers to build sustainable and independent lives. Our findings demonstrate that recovery extends beyond pharmacotherapy, covering the desire for people with psychosis to regain their sense of independence. Evidence has shown a significant link between self-efficacy, self-esteem and hopes to recover in schizophrenia patients [32–34]. Findings from this study suggest love and care, self-efficacy, self-reliance and autonomy are widely desired among PWLE as recovery precursors. This is echoed in similar studies on recovery among individuals with serious mental illnesses (SMIs). Rensburgh & Brooke-Summer discussed how intersectoral and multisectoral approaches enable recovery for people with SMIs in low-and middle-income countries. These include the availability of community support systems, community spaces for recovery, and social spheres of care [30]. Other domains that describe recovery from SMIs include social recovery, which concerns individuals gaining social status and feeling included in society; economic recovery, which deals with individuals being able to sustain their daily lives without depending on their guardians; and individual recovery, which leans more toward achieving normalcy by individuals with SMIs [35]. Community-Based Rehabilitation (CBR) services have also proven effective in supporting recovery from SMIs. However, in Malawi, like most LMICs, available CBR services do not provide adequate skill-acquisition opportunities to support PWLE hands-on skills that will support recovery [1]. This underscores the need for mental health services to not only focus on clinical recovery but also promote the autonomy and empowerment of PWLE by considering the intersectoral and multisectoral approaches. Such interventions include vocational training and peer support programs to foster self-efficacy among PWLE [36], and the use of lay mental health workers, peer support workers, and community-based psychosocial rehabilitation [37,38]. Studies in high-income settings have also reported on the efficacy and effectiveness of these interventions in mental illness and substance use disorders [39]. By challenging the stigmatized identity imposed by society, such programs help restore agency and social value among PWLE – a critical step in reversing what Goffman describes as the internalization of a spoiled identity [23].

The theme of access to healthcare services reveals the significant challenges that PWLE and caregivers encounter in managing psychosis. The availability of nearby healthcare facilities and reliable access to medication are crucial in supporting recovery and reducing the caregiving burden. However, long distances to health facilities and frequent stockouts of essential medicines undermine effective care, contributing to relapses and discouraging patients from seeking help. Addressing these barriers is necessary to ensure equitable access to healthcare, improve patient outcomes, and support caregivers in their critical role. Our findings echo other studies with similar challenges exacerbated by poverty, and the long-term nature of mental illnesses [34,35,40,41]. Furthermore, our findings reflect on the growing systemic inequalities within the healthcare system, where mental health is not given the same priority when it comes to procuring medicines compared to other conditions like HIV/AIDS, TB, and other Non-Communicable Diseases. This underscores the need to fully integrate mental health in primary healthcare frameworks and allocate resources more equitably. Neglect within the health system also reflects institutional stigma – a concept rooted in stigma theory – where societal institutions mirror and reinforce prejudices that PWLE of psychosis face in everyday life.

This study also highlights how photovoice can foster collaboration and inclusion of PWLE to support their recovery and improve mental health systems and outcomes. Participants shared their experiences of what matters most in managing their illness and what changes they believe are necessary, offering valuable insights for shaping more responsive mental health interventions. Our findings align with existing evidence that underscores the importance of collaboration and participation in designing health interventions, particularly in low-resource settings. Similar studies in LMICs have demonstrated how participatory approaches can enhance the acceptability and feasibility of mental health interventions [42], as well as the role of service user empowerment in strengthening mental health systems [43]. A situation analysis conducted by the Support, Comprehensive Care and Empowerment of People with Psychosocial Disabilities in sub-Saharan Africa (SUCCEED) project in Nigeria, Sierra Leon, Malawi, and Zimbabwe recommended the need for user-led research and co-production of mental health interventions especially in low- and middle-income countries where it is currently less common. The situation analysis further notes that involving PWLE and caregivers as equal partners in mental health decision making processes has the potential to promote social inclusion and combat discrimination [1]. However, when participation is done meaningfully, it can counteract social stigma by giving visibility, voice, and dignity to people whose identities have been socially devalued – a core principle of the stigma theory.

Bates et al. also highlighted the benefits of patient involvement in palliative care research, demonstrating how bottom-up interventions in cancer can improve outcomes [44]. Similarly, evidence from mental health research suggests that empowering participants to voice their concerns and co-produce interventions can significantly contribute to their recovery process [5]. In our study, one of the key findings was the role of love and care in recovery, primarily reflected in the formation of supportive relationships. Both PWLE and caregivers emphasised the importance of holistic approaches to psychosis care, extending beyond individual and clinical recovery to address community and household-level support systems. This finding aligns with social support theories, which propose that emotional, instrumental, and informational support are essential for mental health recovery and help mitigate the adverse effects of stigma [45,46]. Lastly, our findings suggest that building strong social networks and engaging in community-level activities can strengthen recovery pathways and improve long-term outcomes for individuals living with psychosis. This also underscores for the integration of caregiver support programs into mental healthcare frameworks, which are currently non-existent. By strengthening social ties and affirming the inherent worth of PWLE, these efforts directly challenge the socially discrediting lables and interactions that are central to Goffman’s framework.

### Implications of our research

Based on this study’s findings, we recommend investing in localized and integrated care models that improve access to mental health services and promote continuous support, especially within community and primary healthcare settings. This involves strengthening Community-Based Rehabilitation (CBR) interventions focused on vocational training and skill development, which can empower people with lived experience of psychosis (PWLE) to regain independence and support their recovery process. Given the severe shortage of mental health professionals in many low- and middle-income countries (LMICs), task-sharing strategies—training non-specialist healthcare workers to provide mental health services—can be crucial in reaching underserved rural communities. Such strategies increase service coverage, enable early detection and referral of identified psychosis cases, and reduce the workload on overstretched specialist services. However, recovery from psychosis goes beyond pharmacotherapy; therefore, mental health policies must also address broader social determinants of mental health like poverty, stigma, and social and economic exclusion. Our study found that stigma operates not only at the personal level but also through institutional and structural barriers that restrict access to economic and social resources. Fully incorporating mental health services into primary healthcare can help normalize mental healthcare, facilitate early diagnosis and intervention, and reduce stigma by making mental health a routine part of healthcare. Additionally, our study highlights the value of participatory methods—especially photovoice—as tools to include marginalized voices in mental health program design and assessment. These approaches enable PWLE and caregivers to share personal experiences, identify obstacles to care, and collaborate with practitioners and policymakers to develop solutions. To ensure long-term effectiveness and relevance, policies and programs must be inclusive and sensitive to local contexts, reflecting the everyday realities of PWLE and caregivers. This requires establishing ongoing mechanisms for their involvement, including representation in policy discussions and incorporating findings from participatory and community-based research.

## Study strengths and limitations

Key strengths of this study include the active involvement of individuals with lived experience and their caregivers, and the high retention rate of recruited participants. The study provided participants with photography training, which proved to be an empowering experience, enabling them to engage with their surroundings critically, enhance their analytical skills, and explore new forms of self-expression [47]. Although this participatory photography method alone may not directly influence social or policy change, it aligns with and has informed the broader goals of the PROMISE project. Specifically, it contributes to the deeper understanding of how psychosis is perceived and experienced by people living with the condition (PWLE) and their caregivers, informed the development of the PROMISE intervention, and promoted narratives of psychosis and recovery.

A fundamental limitation of this study is that the sample was relatively diverse, with specific sub-groups such as adolescents and older people being under-represented. This prevented us from drawing specific conclusions regarding demographic variations. Future research should, therefore, aim at targeted recruitment strategies to include under-represented sub-groups to gain a broader understanding of their narratives, perceptions, and experiences [48]. Participants’ literacy and education levels are other limitations of our study. Although these factors did not prevent participantsfrom making essential contributions through their photos and narratives, this limitation restricted their capacity to participate more actively in the data analysis and interpretation phases or co-authoring the final write-up. This constraint may have narrowed the depth of participant-driven insights and limited opportunities for shared ownership of the research outcomes. Another limitation of this study is the exclusion of individuals with more severe psychosis, adolescents, and the elderly. This resulted in missing perspectives of those with more complex needs or lower levels of functioning and are often not fully represented. This also limited the breadth of insights into the diverse experiences of people living with psychosis. Future research should explore inclusive approaches that enable participation of individuals across the full spectrum of psychosis experiences.

## Conclusion

Individuals living with psychosis and their caregivers desire the elimination of social and economic exclusion to improve recovery and caregiving processes. Love, care, autonomy, and easy access to healthcare are essential precursors to combating social, financial, and systemic exclusion and providing optimal caregiving and recovery. This underscores the need for targeted community-based interventions to eliminate stigma and discrimination, integrated healthcare services, and task-sharing strategies to improve access to mental healthcare services at the community level. Furthermore, a strengthened policy environment around exclusion experienced by PWLE and other systemic barriers can also support improving the welfare of PWLE and caregivers to foster high-quality outcomes for mental healthcare. This calls for an intersectoral and multisectoral approach, part of which is the PROMISE projects’ goal – to enhance psychosis services and promote engagement to improve recovery and reduce treatment gaps.

Furthermore, scaling participatory methods like photovoice in LMICs could help uncover culturally specific insights into challenges of psychosis recovery, empower marginalised groups, and inform the development of inclusive policies and interventions. Expanding these methods to address diverse populations and PWLE of psychosis would enhance their potential to transform research and practice in resource-constrained settings. Their involvement through participatory methods has the potential to create more inclusive and responsive mental health systems.

## Supporting information

S1 Checklist COREQ_Checklist. (DOC)
